# Kaposi Sarcoma: Retrospective Clinical Analysis with a Focus on Age and HIV Serostatus

**DOI:** 10.3390/v18010144

**Published:** 2026-01-22

**Authors:** Zuhal Erçin, Mehtap Toprak

**Affiliations:** 1 Department of Dermatology, Sultan Abdulhamid Han Training and Research Hospital, University of Health Sciences, Istanbul 34668, Turkey; 2 Department of Pathology, Sultan Abdulhamid Han Training and Research Hospital, University of Health Sciences, Istanbul 34668, Turkey; dr.mehtapguner@hotmail.com

**Keywords:** Kaposi sarcoma, HIV, age, epidemiology, comorbidity

## Abstract

Studying the incidence of Kaposi sarcoma in relation to key variables can guide targeted research and subtype-specific clinical interventions. We reviewed the records of all patients who visited our hospital’s dermatology outpatient clinic, and patients who were clinically and histopathologically diagnosed with Kaposi sarcoma were included in the study. The age, gender, lesion location, anti-HIV test results, and comorbidities of the patients were recorded. Thirty-three patients with Kaposi sarcoma were identified. The male/female ratio was 2.7:1. The Kaposi sarcoma lesions were statistically significantly more prevalent in the lower extremities of HIV-negative patients (*p* = 0.005). Receiver operating characteristic (ROC) curve analysis identified 59 years as the optimal age cutoff for distinguishing between HIV-positive and HIV-negative patients. Anti-HIV positivity was significantly higher in individuals aged 59 and younger compared to those aged 60 and older (*p* < 0.001). To the best of our knowledge, this is the first study to demonstrate a statistically significant higher prevalence of lower extremity lesions among HIV-negative patients and to identify 59 years as the optimal age cutoff for distinguishing between HIV-positive and HIV-negative Kaposi sarcoma patients using ROC curve analysis. The age-related patterns observed in this study warrant further investigation.

## 1. Introduction

Kaposi sarcoma, first described by Hungarian dermatologist Moritz Kaposi in 1872, is a multifocal, endothelial proliferation of low-grade malignant potential caused by human herpesvirus 8 (HHV-8), most often with cutaneous involvement with or without visceral extension. There are five distinct subtypes: classic, endemic, iatrogenic, AIDS (acquired immunodeficiency syndrome)-associated (epidemic), and the newest subtype, arising in MSM (men who have sex with men) without HIV (human immunodeficiency virus) infection. Cutaneous lesions are typically dark blue or purple macules, papules, or plaques [1]. Bullous Kaposi sarcoma has also been reported as an uncommon variant [2]. Lymph nodes, mucosae, and viscera may be involved without skin involvement [1]. Kaposi sarcoma can also, though rarely, involve the nail unit [3]. Although HHV-8 is considered the causative agent, multiple co-factors are required, the most powerful of which is HIV co-infection, which elevates the risk up to 20,000-fold. All forms of Kaposi sarcoma are associated with HHV-8 infection as the etiological agent [1]. Immunohistochemically, monoclonal antibodies against LANA (latency-associated nuclear antigen) are routinely used for specific HHV-8 detection [4]. Discovered in 1994 and originally termed Kaposi sarcoma herpesvirus, this gammaherpesvirus is easily transmitted through saliva and blood [1]. Transmission of the virus can also be sexual and may affect MSM. HHV-8 may also be transmitted by solid organ transplantation [5]. HHV-8 may additionally be transmitted via vertical transmission [4]. The role of HHV-8 in the pathogenesis of Kaposi sarcoma appears to be related to some of the proteins produced by the virus. The HHV-8 gene encodes proteins that inhibit retinoblastoma and p53 tumor suppressor genes. HHV-8 viral interferon regulatory factor prevents interferon from repressing the c-myc oncogene. The cytokine viral interleukin-6 produced by HHV-8 causes the increased expression of vascular endothelial growth factor [6]. Thus, HHV-8 supports both angiogenesis and cell proliferation. Other factors affecting tumorigenesis include hypoxia, epigenetic modifications, immunosuppression, and hyperglycemia [7]. In HIV-infected cases, the Tat protein of the HIV virus induces various cytokines that synergistically interact with the products of HHV-8, which may account for the development and progression of Kaposi sarcoma [8]. Kaposi sarcoma in people living with HIV is considered a sign that HIV has developed into AIDS [9]. However, some HIV patients can develop Kaposi sarcoma while having a normal CD4 cell count [10]. HIV contributes to the pathogenesis of Kaposi sarcoma by inducing the immunosuppression necessary for the clinical expression of opportunistic disease [11]. After an acute infection, HHV-8 establishes the latent infection with low gene expression and no production of virions. When latency is disrupted, HHV-8 is reactivated and switches to lytic replication, increasing the gene expression, including for genes with oncogenic and angiogenic properties. Various triggers have been shown to switch HHV-8 from latency to lytic reactivation, such as immunosuppression, unbalanced pro-inflammatory cytokines, and viral co-infections. Studies have shown that decreased levels of T cells are associated with spontaneous lytic production, and low CD4+ T cell levels in HIV-positive patients are associated with the development of Kaposi sarcoma [12].

SARS-CoV-2 (severe acute respiratory syndrome coronavirus 2) infection has also been reported as a possible etiological factor [12,13]. SARS-CoV-2 may cause the transition of latent HHV-8 infection into lytic replication by stimulating the release of pro-inflammatory cytokines with a possible key role of IL-6 [12]. In skin biopsies of an immunocompetent patient with disseminated Kaposi sarcoma following COVID-19, both HHV-8 and SARS-CoV-2 were detected by transmission electron microscopy. An experimental laboratory study examining the effect of SARS-CoV-2 proteins and some anti-COVID-19 drugs on HHV-8 revealed the capacity of both SARS-CoV-2 and azithromycin to manipulate intracellular signaling pathways toward a reactivation of HHV-8 and thereby favoring HHV-8 related diseases such as Kaposi sarcoma [13].

The genetic basis for the ethnogeographic predisposition of Kaposi sarcoma in the classic and endemic subtypes is unclear at present. The contribution of additional exposures has been suggested. These include exposure to quinine, nitrile inhalants, angiotensin-converting enzyme inhibitors, and volcanic soil silicates [1].

The management of Kaposi sarcoma depends on the clinical subtype. In localized cutaneous disease, excision and cryotherapy can be used. Kaposi sarcoma is highly radiosensitive, with complete responses in up to 93% of patients. Liposomal doxorubicin and paclitaxel are approved by the United States Food and Drug Administration as first- and second-line treatments, respectively, for advanced Kaposi sarcoma. There have also been reports of responses to imatinib, sorafenib, bortezomib, nivolumab, and pembrolizumab [1]. Intralesional cidofovir, intralesional vinblastine, intralesional vincristine, photodynamic therapy, Nd-YAG (neodymium-doped yttrium aluminum garnet) laser treatment, topical sirolimus, topical imiquimod, topical alitretinoin, electrochemotherapy, topical and oral propranolol, acitretin, and metformin have also been reported as successful treatments in the literature [4,14,15,16,17,18,19,20,21]. Interferon alfa, although effective in some patients, tends to be associated with lower response rates than paclitaxel and liposomal doxorubicin [22].

Investigating the incidence of Kaposi sarcoma according to various factors can contribute to more targeted research and intervention. This study investigates the epidemiological and clinical features of patients with Kaposi sarcoma at a major teaching hospital in Istanbul. The aim of this study is to examine the incidence and clinical characteristics of different forms of Kaposi sarcoma, with particular emphasis on age patterns and HIV serostatus.

## 2. Materials and Methods

### 2.1. Ethical Committee Approval

The study was conducted in accordance with the Declaration of Helsinki, and the protocol was approved by the Ethics Committee of Istanbul Medipol University with the protocol code E-10840098-202.3.02-3368 on 28 May 2025.

### 2.2. Patient Cohort

A retrospective cohort study design was used. The outpatient records of all patients who visited the dermatology outpatient clinic of our hospital between October 2016 and March 2025 were reviewed, and patients who were clinically and histopathologically diagnosed with Kaposi sarcoma were included in the study. Our hospital was a military hospital before October 2016, but it became a state hospital in October 2016 and currently serves as a tertiary referral center. For this reason, outpatient clinic records have been available for review since October 2016. Outpatient clinic records were screened for Kaposi sarcoma using the ICD (International Classification of Diseases) code C46. The age, gender, lesion location, anti-HIV test results, and comorbidities of patients diagnosed with Kaposi sarcoma were recorded.

### 2.3. HIV ECLIA

Anti-HIV screening was performed using an electrochemiluminescence immunoassay (ECLIA) on the Roche cobas e 801 system with the Elecsys HIV Duo kit (Roche Diagnostics GmbH, Mannheim, Germany).

### 2.4. HIV Immunochromatographic Assay

Samples with reactive HIV screening results were confirmed using an HIV-1/2 antibody (Ab) differentiation immunoassay (Geenius HIV-1/2 Supplemental Assay, Bio-Rad Laboratories, Redmond, WA, USA).

### 2.5. Histopathological Processing and Immunohistochemistry

Formalin-fixed, paraffin-embedded tissue sections were stained with hematoxylin and eosin (H&E) following standard histopathological procedures and examined using light microscopy for morphological assessment. Histopathological diagnoses were confirmed by positive immunohistochemical staining for HHV-8. For each case, at least one representative paraffin block was selected for immunohistochemical analysis. Immunohistochemistry was performed on formalin-fixed, paraffin-embedded tissue samples using 3 µm thick sections. Tissue sections were mounted onto electrostatically charged slides and dried at 70 °C for at least 1 h. The entire immunohistochemical staining process, including deparaffinization and antigen retrieval, was performed using a fully automated immunohistochemistry platform (Leica BOND-MAX Detection System; Leica Biosystems, Melbourne, Australia). Signal detection was performed using a biotinylated horseradish peroxidase (HRP) multimer-based detection system with hydrogen peroxide as the substrate and a ready-to-use 3,3′-diaminobenzidine (DAB) tetrahydrochloride chromogen kit. The primary antibody used was HHV-8 (Leica Biosystems, clone 13B10; dilution 1:50), applied according to the manufacturer’s instructions. Following immunostaining, slides were counterstained using a Leica XL automated stainer and mounted with a Leica CV5030 automated coverslipper (Leica Biosystems, Nussloch, Germany).

### 2.6. Statistical Analysis

The data obtained from the study were analyzed using IBM SPSS Statistics for Windows, version 30.0 (IBM Corp., Armonk, NY, USA). Descriptive statistics for the sociodemographic and clinical characteristics of patients with Kaposi sarcoma were presented as counts (*n*), percentages (%), means, and standard deviations. Categorical variables were analyzed using Fisher’s exact test. The Shapiro–Wilk test was used to assess the normality of the age at diagnosis variable, and its association with anti-HIV positivity was evaluated using the Mann–Whitney U test. In addition, to identify the optimal age at diagnosis that could be a predictor for anti-HIV positivity, a receiver operating characteristic (ROC) curve analysis was performed, and the Youden index was used. A *p*-value of <0.05 was considered statistically significant.

## 3. Results

**Figure 1:**
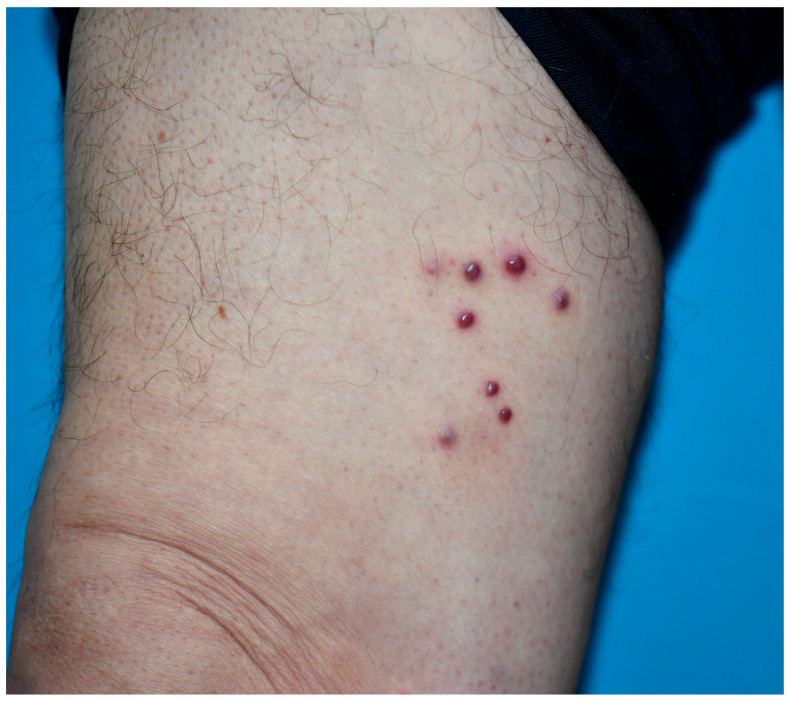
Kaposi sarcoma lesions on the right leg of a 74-year old man.

**Figure 2:**
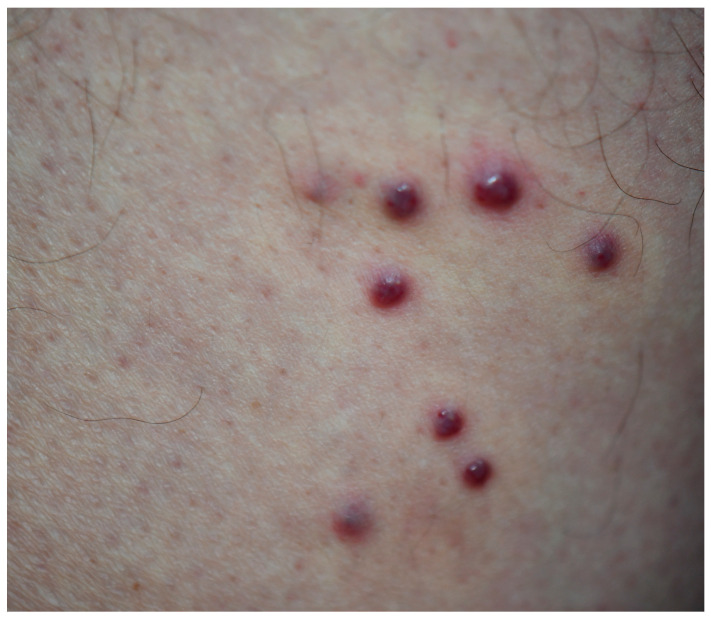
Close-up view of Kaposi sarcoma lesions on the right leg of a 74-year old man.

**Figure 3:**
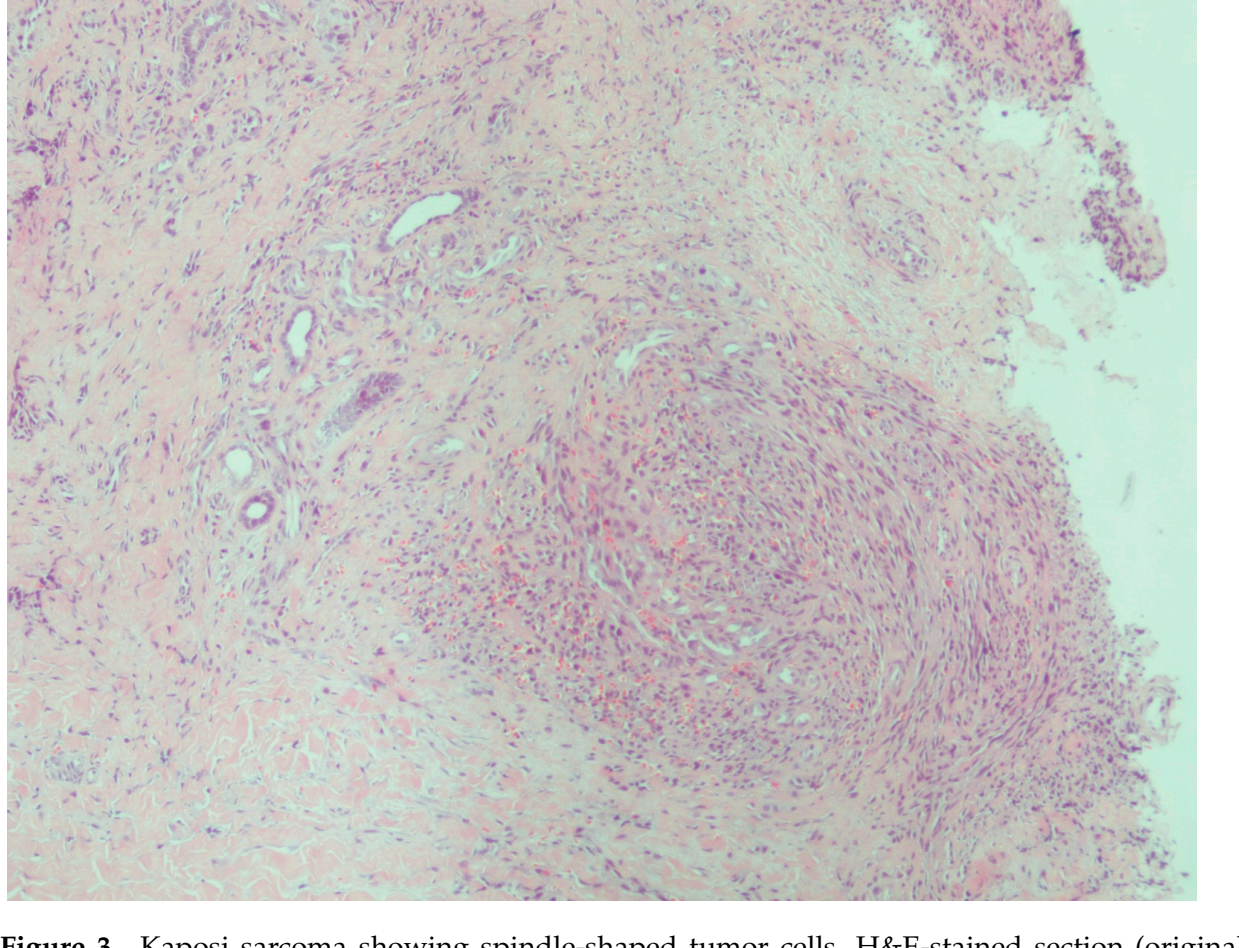
Kaposi sarcoma showing spindle-shaped tumor cells, H&E-stained section (original magnification, ×10).

**Figure 4:**
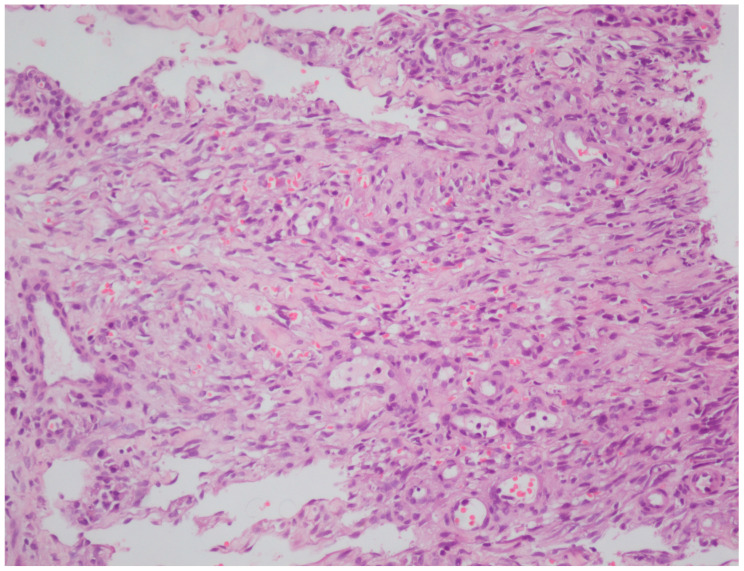
Kaposi sarcoma showing vascular slits filled with erythrocytes, H&E-stained section (original magnification, ×20).

**Figure 5:**
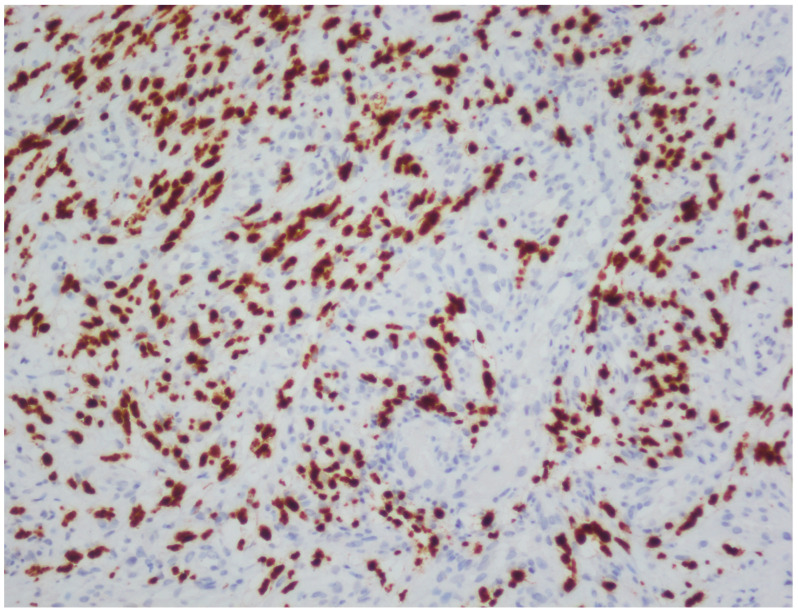
HHV-8 immunohistochemistry in HIV-positive Kaposi sarcoma (original magnification, ×20).

**Figure 6:**
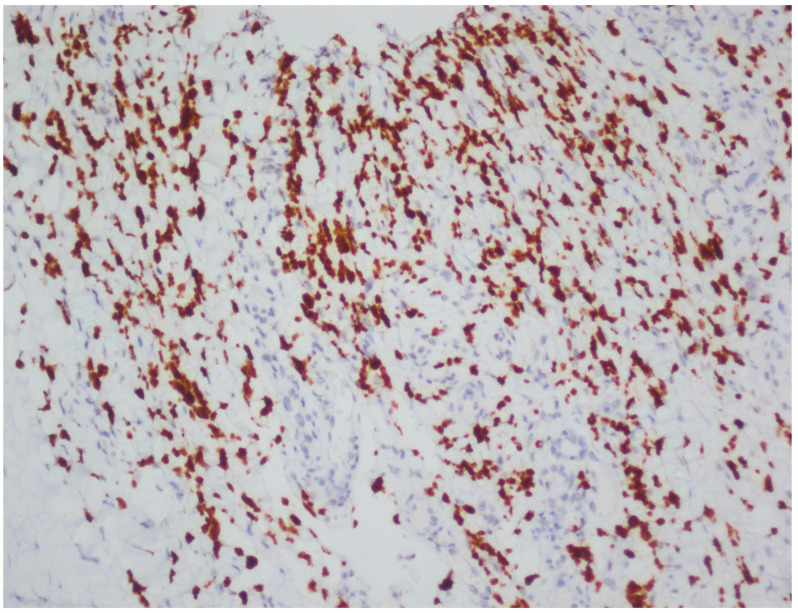
HHV-8 immunohistochemistry in HIV-negative Kaposi sarcoma (original magnification, ×20).

**Figure 7:**
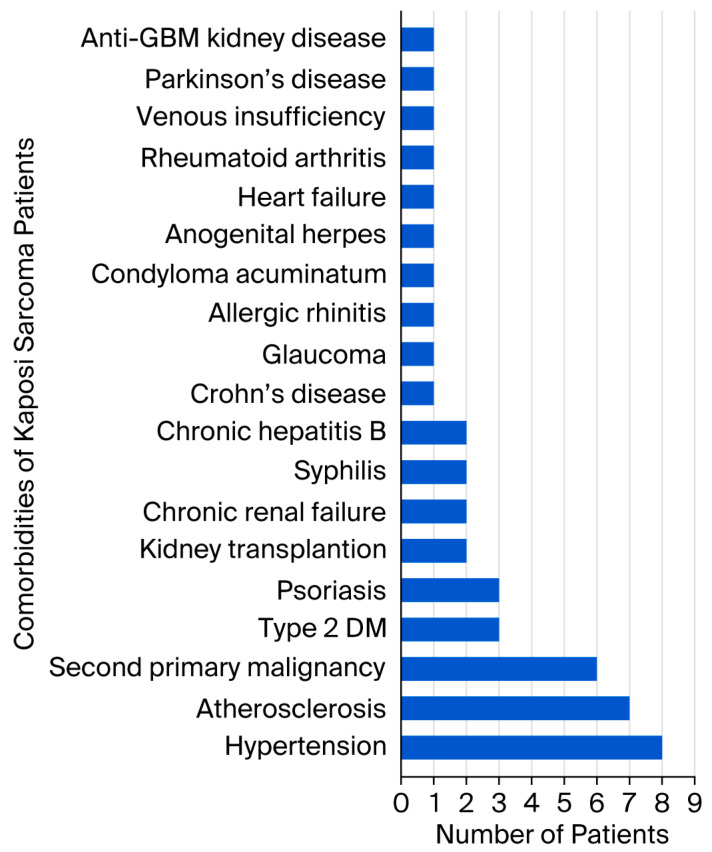
Comorbidities observed in patients with Kaposi sarcoma.

**Figure 8:**
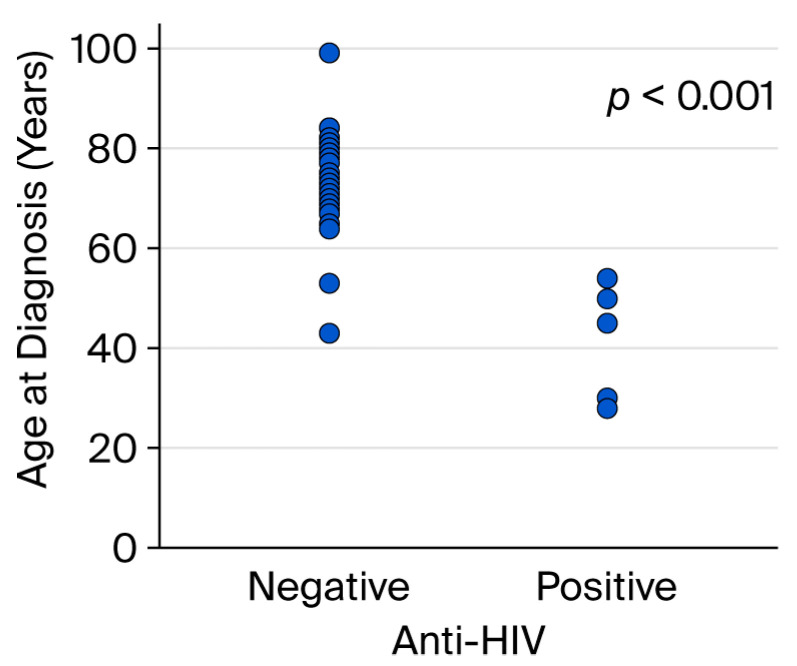
Comparison of the mean age at diagnosis of Kaposi sarcoma by HIV serostatus.

**Figure 9:**
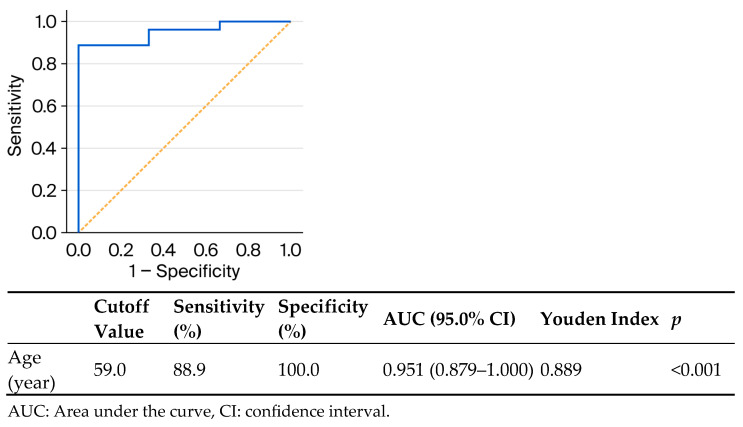
ROC curve evaluating the performance of age at diagnosis in predicting HIV positivity among patients with Kaposi sarcoma. The solid blue line represents the ROC curve, and the dashed orange diagonal line indicates the reference corresponding to random classification (AUC = 0.5). The area under the curve (AUC) was 0.951 (95% CI: 0.879–1.000), indicating excellent discriminatory ability. The optimal cutoff age, determined using the Youden index (0.889), was 59 years.

All patients who visited our outpatient clinic between October 2016 and March 2025 were screened, and 33 patients with Kaposi sarcoma were identified. These 33 patients underwent a punch biopsy and had been clinically (Figure 1 and Figure 2) and histopathologically diagnosed with Kaposi sarcoma (Figure 3 and Figure 4). All patients were screened for HIV, and reactive samples underwent confirmatory testing. Anti-HIV test results of the patients are summarized in Table 1. The histopathological diagnoses were confirmed by positive immunohistochemical staining for HHV-8. Immunohistochemical staining for HHV-8 revealed different staining patterns between the HIV-positive and HIV-negative Kaposi sarcoma cases. HHV-8 immunohistochemistry in HIV-positive Kaposi sarcoma demonstrated strong, dark brown nuclear staining in a high proportion of spindle tumor cells, with diffuse and relatively homogeneous positivity, high density of HHV-8-positive cells, and frequent contiguous clusters of labeled nuclei, consistent with a high viral antigen burden. HHV-8 immunohistochemistry in HIV-negative Kaposi sarcoma demonstrated predominantly moderate to weak nuclear staining compared with the HIV-positive case, with more patchy and heterogeneous positivity, intermixed negative areas, a lower density of HHV-8-positive nuclei, increased visibility of unstained stromal cells, and more scattered positive cells with fewer densely packed clusters. These findings indicate a relatively reduced HHV-8 immunoreactivity in the HIV-negative Kaposi sarcoma compared with the HIV-positive case (Figure 5 and Figure 6). Patients were screened for metastasis in the medical oncology department. Only one patient (3%) had metastasis to the gastrointestinal system. This patient was HIV-negative and had been receiving systemic treatment with 16 mg/day methylprednisolone for three months for anti-glomerular basement membrane (anti-GBM) disease of the kidney. The age, gender, anti-HIV test results, lesion locations, and comorbidities of the patients are summarized in Table 2. Twenty-four (72.7%) of the patients were male, and nine (27.3%) were female. The male/female ratio was 2.7:1. The patients’ ages ranged from 28 to 99, with a median age of 72. The mean age and standard deviation of the patients was 67.61 ± 15.99. The anti-HIV test result was negative in 81.8% of cases (*n* = 27), and positive in 18.2% of cases (*n* = 6). Lesion sites were grouped into upper extremities, lower extremities, head, trunk, diffuse skin involvement, and gastrointestinal involvement, and the relationship between anti-HIV test results and lesion sites was examined (Table 3). This examination revealed that lesions were statistically significantly more prevalent in the lower extremities of HIV-negative patients (*p* = 0.005). Additionally, HIV-positive patients were statistically significantly more likely to have widespread skin involvement (*p* = 0.004) and trunk involvement (*p* = 0.028). No statistically significant difference was found between gender and lesion location (*p* = 0.999). In 24.2% of cases (*n* = 8), no comorbidities were present, while at least one comorbidity was present in 75.8% of cases (*n* = 25). The comorbidities of the patients are summarized graphically in Figure 7. There was a statistically significant difference in the mean age between HIV-positive and HIV-negative patients (*p* < 0.001) (Figure 8). Receiver operating characteristic (ROC) curve analysis shows that 59 years is the cutoff value for age between HIV-positive and HIV-negative patients (Figure 9). After dividing and analyzing the groups according to the optimal cutoff value calculated from the ROC curve, anti-HIV positivity was found to be significantly higher among individuals aged 59 or younger than among those aged 60 or older (*p* < 0.001) (Table 4).

## 4. Discussion

The current study is a retrospective analysis of 33 patients with Kaposi sarcoma who were treated at our institution between the years 2016 and 2025. Twenty-four (72.7%) of the patients were male, and nine (27.3%) were female. The predominance of men in this cohort is in line with the literature; Kaposi sarcoma can occur at any age and in both sexes but is more common in adult men. The incidence of Kaposi sarcoma is 0.5 in men and 0.3 in women per 100,000 people [23]. In our study, the male/female ratio was 2.7:1. The overall male/female ratio in patients with Kaposi sarcoma appears to range from 2.5:1 to 9:1 [22]. The higher prevalence of Kaposi sarcoma in men than in women is likely due to various contributing factors, including hormonal, viral, and genetic factors, as well as high-risk behavior [24]. The predominance of males may additionally be explained by the role of sex steroids in the immune system and by females having higher levels of circulating IgG, IgM, and CD4+ T cells [25]. In our study, all of the six HIV-positive patients were male, which is likely due to high-risk behavior in men. This finding is consistent with that reported in a study from Taiwan [26]. The mean age and standard deviation of the patients in our study was 67.61 ± 15.99. This result is quite similar to that of another study conducted in our country, in which the mean age was found to be 68.42 ± 12.80 years [25]. The study conducted in Taiwan found a lower mean age of 58.7 ± 21.2 [26]. The median age of our patients was 72. A study conducted in Finland found that the median age of patients was 44.5 years old [10], and in another study conducted in China, the median age was found to be 60 [7]. Both the mean age and median age of our patients were found to be higher than in other countries in different geographical regions. The reason for this is probably that classic Kaposi sarcoma, which is usually seen in older age groups, is more common in our country than other subtypes and occurs predominantly in men of Eastern European and Mediterranean origin [4].

In our study, a statistically significant higher prevalence of lesions was observed in the lower extremities among HIV-negative patients (*p* = 0.005). Previous retrospective studies on Kaposi sarcoma have reported the lower extremities as the most common site of lesions [10,11,25]. The study conducted in Taiwan found that HIV-positive patients had a lower incidence of lower extremity lesions compared to HIV-negative patients [26]. To the best of our knowledge, this is the first study to demonstrate a statistically significant higher prevalence of lesions in the lower extremities among HIV-negative patients. Why Kaposi sarcoma is common on the lower limbs is not clearly understood [11]. It has been suggested that lymphedema may contribute to tumorigenesis via local immunosuppression [25]. Some environmental factors such as exposure to volcanic soils have been hypothesized in the pathogenesis of Kaposi sarcoma. Chronic exposure of the skin to iron or alumino-silicate might induce localized immune dysfunction, which might provide an additional explanation for the topography of the lesions at the extremities of the body [27]. In our study, a statistically significant association was observed between HIV-positive status and widespread skin lesion distribution in Kaposi sarcoma patients. This finding was also reported in a study conducted in China [7]. HIV-positive patients in our study were statistically significantly more likely to have trunk involvement, which was also observed in a study conducted in Taiwan [26]. No statistically significant difference was found between gender and lesion location. The same finding was observed in a study conducted in Cameroon [28].

In our study, no comorbidities were observed in 24.2% of our cases (*n* = 8), while 75.8% of our cases (*n* = 25) presented with at least one comorbidity. The most common comorbidity in our study was hypertension (*n* = 8, 24.2%), followed by atherosclerosis (*n* = 7, 21.2%). Hypertension was probably the most common comorbidity in our study because the mean age of our patients was over 65. A study found that blood pressure increases with age, with an average increase of 6.4 mmHg in systolic blood pressure per decade [29]. The second-most common comorbidity in our study was atherosclerosis. Atherosclerosis may have contributed to the development of Kaposi sarcoma by affecting tumorigenesis through hypoxia. In six patients (18.2%), a second primary malignancy had been diagnosed before the onset of Kaposi sarcoma. Two patients had a history of prostate carcinoma, and one patient had a history of both papillary thyroid carcinoma and glioblastoma. Another patient had a history of renal cell carcinoma, one had diffuse large B cell lymphoma, and one had basal cell carcinoma. All six patients were male, HIV-negative, and aged 69 years or older. The prevalence of second primary malignancies in patients with Kaposi sarcoma ranges from 8.6% to 40% in the literature [10]. An 18.2% occurrence of second primary malignancies was observed in our cohort, consistent with the existing literature. The literature highlights a notable association between Kaposi sarcoma and second primary malignancies, particularly those affecting the lymphoreticular system [30]. In our study, three patients had type 2 diabetes mellitus, and three had psoriasis. The relationship between Kaposi sarcoma and psoriasis is controversial. According to one study, the risk of Kaposi sarcoma among patients with psoriasis is comparable to that in the general population, with no apparent interaction between the two conditions [31]. However, diabetes mellitus has been identified in the literature as an important predisposing factor for the development of Kaposi sarcoma, with the high-glucose microenvironment suggested as a contributing mechanism [32]. In our study, two patients had undergone kidney transplantation, two had chronic renal failure, two had a history of syphilis, and two had chronic hepatitis B. Single-occurrence comorbidities observed in the cohort included Crohn’s disease, glaucoma, allergic rhinitis, condyloma acuminatum, anogenital herpes, heart failure, rheumatoid arthritis, chronic peripheral venous insufficiency, Parkinson’s disease, and anti-glomerular basement membrane kidney disease. The patients diagnosed with syphilis, condyloma acuminatum, and anogenital herpes were concurrently HIV-positive. The literature describes a case of disseminated Kaposi sarcoma in an HIV-negative individual with chronic hepatitis B infection [26]. Reports in the literature have also documented cases of Kaposi sarcoma in patients undergoing hemodialysis or peritoneal dialysis for chronic renal failure [33,34]. However, our patients with chronic renal failure were not undergoing hemodialysis or peritoneal dialysis. No documented association exists between Parkinson’s disease, glaucoma, allergic rhinitis, and Kaposi sarcoma. Chronic peripheral venous insufficiency may have contributed to the development of Kaposi sarcoma through chronic lymphedema, whereas heart failure may have contributed through tissue hypoxia.

Iatrogenic Kaposi sarcoma was first described in 1978 in a renal transplant recipient and was found to be related to a reactivation of HHV-8 in immunosuppressed transplant patients [25,27]. Additionally, HHV-8 may be transmitted through donor organs [27]. Kaposi sarcoma has been reported to be 200 times more frequent in transplant recipients than in the general population. In the cases of non-transplant-related iatrogenic Kaposi sarcoma, the most common causative drugs are corticosteroids and cyclosporine [22]. Typically, steroid-associated immunosuppression has been considered when the dose exceeds 20 mg prednisone/day, particularly in chronic use [35]. In our study, all HIV-positive patients were aged 54 years or younger. Only three HIV-negative patients were under the age of 54, two of whom had undergone kidney transplantation and one of whom had been receiving 16 mg/day of methylprednisolone, which is equivalent to 20 mg/day of prednisone for three months, to treat anti-GBM kidney disease. These three patients may be classified as having iatrogenic Kaposi sarcoma. The patient with Crohn’s disease was a 68-year-old HIV-negative man receiving treatment with mesalazine and azathioprine. The patient with rheumatoid arthritis was an 82-year-old HIV-negative woman receiving treatment with 12.5 mg/week methotrexate and 5 mg/day prednisolone. However, whether these two patients should be classified as having classic or iatrogenic Kaposi sarcoma remains a matter of debate. Based on current knowledge, we classified these two patients as having classical Kaposi sarcoma. Consequently, in our cohort, 24 patients had classical, 3 had iatrogenic, and 6 had HIV-associated Kaposi sarcoma. Further studies are needed to clearly identify which immunosuppressive drugs and dosages are associated with the development of non-transplant-related iatrogenic Kaposi sarcoma.

In our study, a statistically significant difference in the mean age was observed between HIV-positive and HIV-negative patients (*p* < 0.001). A similar finding was reported by Chalya et al., who observed that patients with AIDS-associated Kaposi sarcoma were significantly younger than those with HIV-negative disease [11]. In the study by Hong and Lee, all HIV-positive patients were under 60 years of age, whereas the HIV-negative group included individuals aged 60 years and older; this difference was statistically significant [8]. All HIV-positive patients in our study were aged 54 years or younger; however, ROC curve analysis identified 59 years as the optimal age cutoff for distinguishing between HIV-positive and HIV-negative patients, demonstrating excellent discriminatory ability (AUC = 0.951), with a sensitivity of 88.9% and specificity of 100.0%. Anti-HIV positivity was significantly more prevalent in the group aged 59 years or younger compared to those aged 60 years or older (*p* < 0.001) when patients were stratified based on the optimal age cutoff of 59 years identified using ROC curve analysis. To the best of our knowledge, this is the first study to identify 59 years as the optimal age cutoff for distinguishing between HIV-positive and HIV-negative patients using ROC curve analysis. This suggests that age may serve as a clinically useful indicator in differentiating between these patient populations. Kaposi sarcoma occurring in individuals aged 60 years and older may, in the future, be classified as a geriatric condition associated with immunosenescence. While the absence of HIV infection may be presumed in such cases, routine HIV testing should not be omitted without sufficient supporting evidence. Further research is needed to confirm this association. The observed age-related patterns warrant further investigation. The limitations of this study are its single-center design and relatively small sample size. Nevertheless, it provides valuable insights from real-world data and lays the groundwork for future research in this field.

## 5. Conclusions

Kaposi sarcoma, first described by Hungarian dermatologist Moritz Kaposi in 1872, is a multifocal, endothelial proliferation of low-grade malignant potential caused by human herpesvirus 8 (HHV-8), most often with cutaneous involvement with or without visceral extension. All forms of Kaposi sarcoma are associated with HHV-8 infection as the etiological agent, but not all forms of Kaposi sarcoma are associated with HIV infection. There are five distinct subtypes of Kaposi sarcoma: classic, endemic, iatrogenic, AIDS-associated (epidemic), and the newest subtype, arising in MSM without HIV infection. This research advances current knowledge by demonstrating that 59 years is the optimal age cutoff for distinguishing between HIV-positive and HIV-negative Kaposi sarcoma patients using ROC curve analysis. Anti-HIV positivity was significantly more prevalent in the group aged 59 years or younger compared to those aged 60 years or older (*p* < 0.001). The observed age-related patterns warrant further investigation.

## Figures and Tables

**Table 1:** HIV status of Kaposi sarcoma patients.

Case	Age	Gender	Anti-HIV	
			ECLIA	HIV-1/2 Ab Differentiation Assay
1	53	Male	Negative	
2	82	Male	Negative	
3	68	Male	Negative	
4	67	Female	Negative	
5	81	Female	Negative	
6	65	Female	Negative	
7	50	Male	Positive	Positive
8	75	Male	Negative	
9	84	Male	Negative	
10	30	Male	Positive	Positive
11	45	Male	Positive	Positive
12	78	Male	Negative	
13	54	Male	Positive	Positive
14	99	Female	Negative	
15	71	Male	Negative	
16	81	Female	Negative	
17	72	Male	Negative	
18	69	Male	Negative	
19	70	Male	Negative	
20	73	Female	Negative	
21	79	Male	Negative	
22	74	Male	Negative	
23	43	Male	Negative	
24	81	Male	Negative	
25	28	Male	Positive	Positive
26	77	Male	Negative	
27	54	Male	Positive	Positive
28	72	Female	Negative	
29	53	Male	Negative	
30	82	Female	Negative	
31	64	Female	Negative	
32	80	Male	Negative	
33	77	Male	Negative	

**Table 2:** Demographic and clinical features of Kaposi sarcoma patients.

Case	Age	Gender	Localization	Anti-HIV	Comorbidity
1	53	Male	Bilateral cruris and feet	Negative	Kidney transplant recipient
2	82	Male	Bilateral wrists and feet	Negative	Hypertension, atherosclerosis
3	68	Male	Left hand, right foot	Negative	Hypertension, chronic renal failure, Crohn’s disease
4	67	Female	Left arm, left hand, right foot	Negative	None
5	81	Female	Right ankle	Negative	Hypertension, glaucoma, chronic peripheral venous insufficiency
6	65	Female	Left foot	Negative	Allergic rhinitis
7	50	Male	Right axilla and chest	Positive	Type 2 diabetes mellitus (DM), condyloma acuminatum, anogenital herpes
8	75	Male	Right cruris	Negative	Type 2 diabetes mellitus, atherosclerosis, renal cell carcinoma
9	84	Male	Right foot	Negative	Hypertension, atherosclerosis
10	30	Male	Extensive skin lesions	Positive	None
11	45	Male	Extensive skin lesions	Positive	None
12	78	Male	Left ankle	Negative	Psoriasis
13	54	Male	Extensive skin lesions	Positive	Syphilis
14	99	Female	Bilateral cruris	Negative	Hypertension, atherosclerosis
15	71	Male	Left hand, left foot	Negative	Diffuse large B cell lymphoma, type 2 DM, atherosclerosis, chronic hepatitis B
16	81	Female	Right knee	Negative	Atherosclerosis
17	72	Male	Left elbow	Negative	Psoriasis
18	69	Male	Left arm, bilateral feet	Negative	Thyroid papillary carcinoma, glioblastoma
19	70	Male	Left knee, left labial commissure	Negative	None
20	73	Female	Right arm, right foot	Negative	Hypertension
21	79	Male	Bilateral cruris	Negative	Basal cell carcinoma
22	74	Male	Right thigh	Negative	Prostate carcinoma
23	43	Male	Bilateral cruris	Negative	Kidney transplant recipient
24	81	Male	Right ankle	Negative	Psoriasis
25	28	Male	Left arm, left cruris, trunk	Positive	None
26	77	Male	Bilateral arms and legs	Negative	Parkinson’s disease, hypertension, prostate carcinoma
27	54	Male	Left cruris, left ankle	Positive	Syphilis
28	72	Female	Right foot	Negative	None
29	53	Male	Bilateral feet, gastric antrum and corpus	Negative	Anti-glomerular basement membrane kidney disease
30	82	Female	Left cruris	Negative	Chronic hepatitis B, rheumatoid arthritis
31	64	Female	Left arm	Negative	None
32	80	Male	Right foot	Negative	None
33	77	Male	Right ankle	Negative	Hypertension, atherosclerosis, chronic renal failure, heart failure

**Table 3:** Anti-HIV test results and lesion locations.

	Anti-HIV		*p* *
	Negative, *n* (%)	Positive, *n* (%)	
Lower extremities	25 (92.6)	2 (33.3)	0.005
Upper extremities	9 (33.3)	1 (16.7)	0.640
Trunk	0 (0.0)	2 (33.3)	0.028
Head	1 (3.7)	0 (0.0)	0.999
Diffuse skin involvement	0 (0.0)	3 (50.0)	0.004
Gastrointestinal system	1 (3.7)	0 (0.0)	0.999

* Fisher’s exact test.

**Table 4:** The relationship between HIV positivity and age at diagnosis with Kaposi sarcoma.

		Anti-HIV		*p*
		Negative	Positive	
Age (year), mean ± standard deviation		73.0 ± 11.2	43.5 ± 11.7	<0.001 ^a^
Age (year), *n* (%)	≤59	3 (33.3)	6 (66.7)	<0.001 ^b^
	≥60	24 (100.0)	0 (0.0)	

^a^ Mann–Whitney U test; ^b^ Fisher’s exact test.

## Data Availability

The original contributions presented in this study are included in the article, in Table 1. Further inquiries can be directed to the corresponding author.

## Abbreviations

The following abbreviations are used in this manuscript:

HHV-8	Human herpesvirus 8
AIDS	Acquired immunodeficiency syndrome
MSM	Men who have sex with men
HIV	Human immunodeficiency virus
SARS-CoV-2	Severe acute respiratory syndrome coronavirus 2
Nd-YAG	Neodymium-doped yttrium aluminum garnet
ICD	International Classification of Diseases
ECLIA	Electrochemiluminescence immunoassay
ROC	Receiver operating characteristic
DM	Diabetes mellitus
AUC	Area under the curve
CI	Confidence interval
LANA	Latency-associated nuclear antigen
Anti-GBM	Anti-glomerular basement membrane
Ab	Antibody
H&E	Hematoxylin and Eosin
HRP	Horseradish peroxidase
DAB	3,3′-diaminobenzidine
